# Symptomatic bilateral massive pulmonary embolism and proximal and distal deep vein thrombosis following arthroscopic meniscus surgery: A case report

**DOI:** 10.1097/MD.0000000000025372

**Published:** 2021-04-02

**Authors:** Sang Hyun Jeon, Geon Ho Kwon, Man Soo Kim

**Affiliations:** a Department of Orthopedic Surgery, Incheon St. Mary's Hospital, College of Medicine, The Catholic University of Korea, Incheon; b Department of Orthopedic Surgery, Seoul St. Mary's Hospital, College of Medicine, The Catholic University of Korea, Seoul, Korea.

**Keywords:** Deep vein thrombosis, meniscus repair, pulmonary thromboembolism, venous thromboembolism

## Abstract

**Rationale:**

The incidence of venous thromboembolism (VTE) after knee arthroscopy is remarkably lower than that of arthroplasty. We describe a patient with symptomatic bilateral massive pulmonary thromboembolism (PTE) and deep vein thrombosis (DVT) in the femoral and popliteal veins after arthroscopic meniscus repair surgery.

**Patient concerns:**

The patient underwent arthroscopic meniscus repair with no intraoperative complication. There were no underlying diseases such as hypertension or diabetes. On day 5 postoperative, the patient complained of dyspnea, chest discomfort, and nausea after standing.

**Diagnosis:**

On DVT and PTE computed tomography, there were multifocal PTEs in the distal portion of the main and segmental branches of both pulmonary arteries. There was a focal thrombosis in the left deep femoral vein, as well as small DVTs in the left popliteal and calf veins.

**Interventions:**

After 3 days of low-molecular-weight heparin 1 mg/kg every 12 hours, treatment was changed to an oral drug, dabigatran, for 6 months.

**Outcomes:**

There were no PTE or DVT findings on computed tomography at 6 months postoperative. The patient did not complain of symptoms related to PTE or DVT at 6 months after the operation, has returned to work, and is living without discomfort.

**Lessions:**

The frequency of VTE is very low after arthroscopic meniscus surgery, but it represents a life-threatening event. Our patient had risk factors for VTE including obesity, surgery time of ∼60 minutes, and immobilization. Although arthroscopic meniscus surgery is relatively safe, evaluation of risk factors for VTE should be performed before and after surgery, and appropriate thromboprophylaxis should be provided when necessary.

## 1 Introduction

Venous thromboembolism (VTE) is common after hip and knee joint arthroplasty,^[1]^ and thromboprophylaxis for VTE prevention in knee and hip joint arthroplasty has been established.^[2–4]^ Knee arthroscopy surgery is one of the most commonly performed orthopedic surgeries in Korea.^[5,6]^ It is a relatively safe operation with a low risk of serious complications. The incidence of VTE is remarkably lower than that with arthroplasty, by 1% to 10%.^[7,8]^ Therefore, a protocol for thromboprophylaxis has not been established. However, if VTE occurs, it is a serious complication and a life-threatening event. Here, we describe a patient with symptomatic bilateral massive pulmonary thromboembolism (PTE) and deep vein thrombosis (DVT) in the femoral and popliteal veins after arthroscopic meniscus surgery.

## 2 Case presentation

### 2.1 Patient information

**Figure 1:**
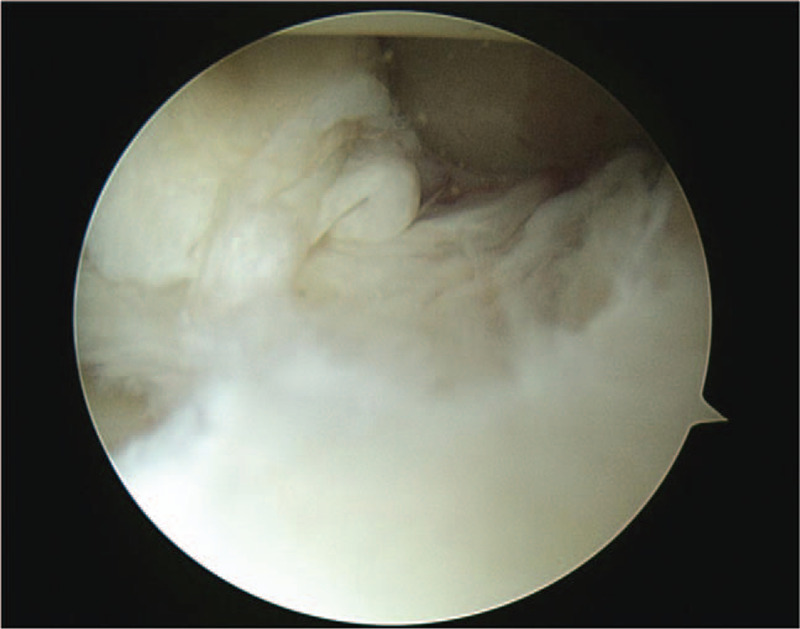
In the lateral meniscus, a complex tear with horizontal and radial tears from the mid body to the posterior horn was observed.

**Figure 2:**
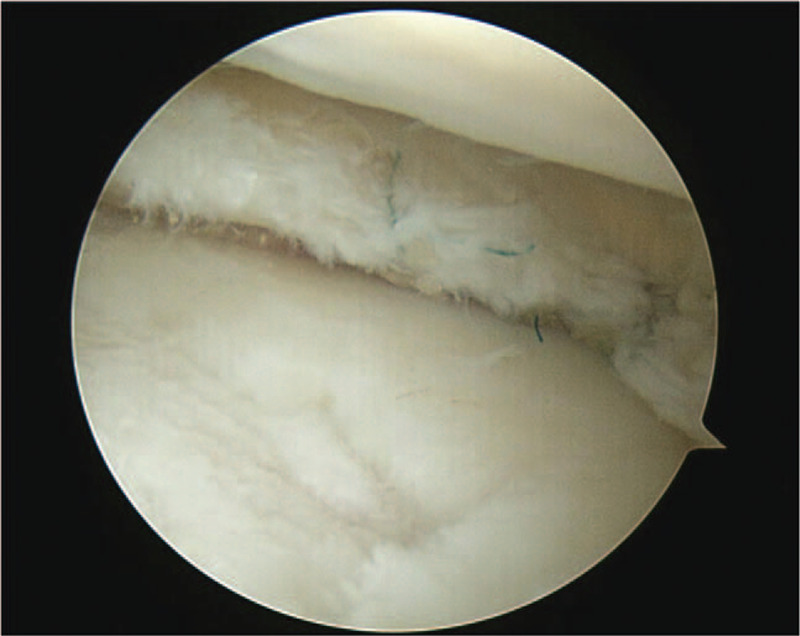
Meniscus repair was performed after marginal debridement of the lateral meniscus using both inside-out and all-inside techniques.

A 32-year-old man visited the outpatient clinic for left knee pain and swelling that occurred after falling on the stairs. On physical examination, there was lateral joint line tenderness, and 40 cc of bloody discharge was aspirated by hemarthrosis. The patient was a courier driver, and he had no history of alcohol or tobacco use. There were no underlying diseases such as hypertension or diabetes. The patient's height was 180 cm, his weight was 118 kg, and his body mass index (BMI) was 36.4, which was class III obesity.^[9]^ On magnetic resonance imaging, a lateral meniscus flap tear was observed, and surgery was scheduled. The operation was performed with tourniquet inflation up to 300 mm Hg under general anesthesia. The patient's obesity was so severe that it was not easy to secure the joint space, and the operation took longer than expected. Tourniquet time was 62 minutes and total operation time was 80 minutes. A complex meniscal tear with horizontal and radial tears from the mid body to the posterior horn was observed (Fig. 1), and repair was performed after marginal debridement using both inside-out and all-inside techniques (Fig. 2). The patient had no comorbidities other than high BMI, no vascular diseases, and did not take antithrombotic drugs or hormones. None of his family members had a history of VTE. After surgery, a long leg splint was applied, and a nonweight bearing position was maintained. Straight leg-raising exercises were performed from the day after surgery to strengthen the quadriceps muscle. Similar to other arthroscopy patients, thromboprophylaxis medications were not administered. The patient was scheduled to be discharged on the second day after surgery, but due to personal reasons, the discharge was changed to day 6. The patient remained in the hospital without any specific symptoms until day 4 after surgery.

### 2.2 Findings

On day 5 postoperative, the patient complained of dyspnea, chest discomfort, and nausea abruptly after standing, and these symptoms were not alleviated after returning to bed. His temperature was 36.7°C, heart rate 95 beats per minute, respiratory rate 20 breaths per minute, blood pressure 80/50 mm Hg, and oxygen saturation (SaO2) 100%. On electrocardiography, there was sinus rhythm but also ST segment elevation on anterior leads. Echocardiogram was performed and showed no significant abnormal signs. On laboratory results, there was increase in fibrinogen and d-dimer, antithrombin III, and fibrinogen degradation production, while cardiac markers were within normal range.

### 2.3 Diagnostic assessment and therapeutic intervention

**Figure 3:**
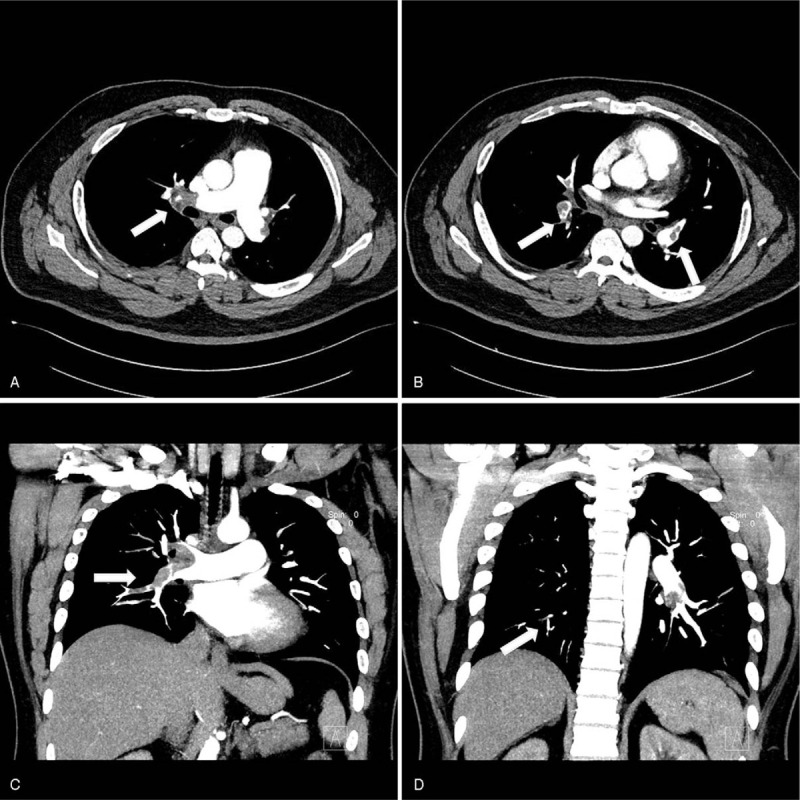
Multifocal PTEs in the distal portion of the main and segmental branches of both pulmonary arteries. Axial view shows a massive PTE in the main pulmonary artery (white arrow) (A), Coronal and axial view shows a PTE in the segmental branches of both pulmonary arteries (white arrow) (B, C, D). PTEs = pulmonary thromboembolisms.

**Figure 4:**
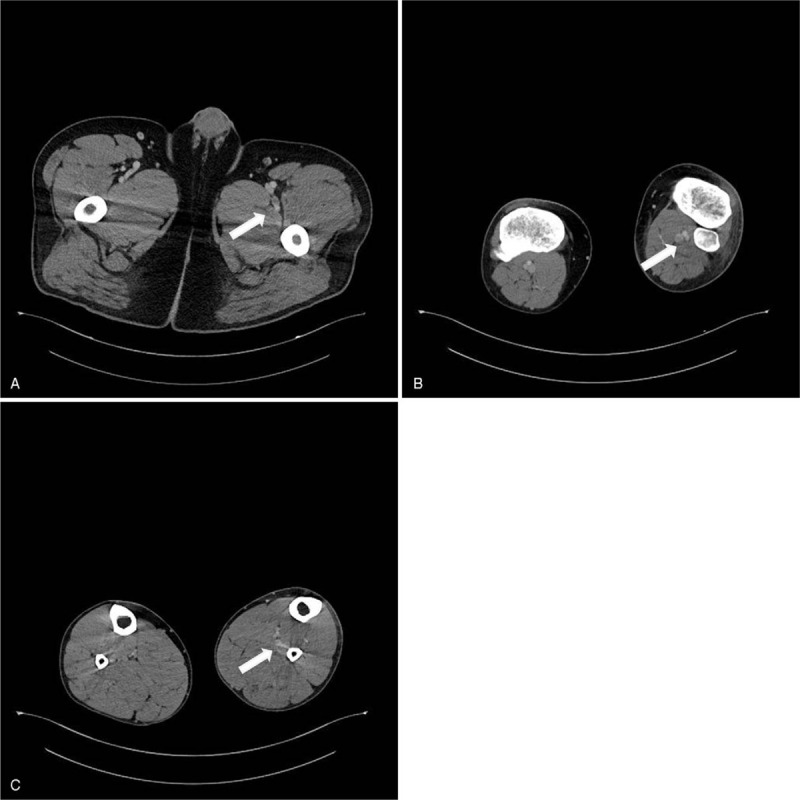
Focal DVTs of the left deep femoral vein (white arrow) (A), small DVTs in the left popliteal and calf veins (white arrow) (B, C). DVTs = deep vein thromboses.

The patient had symptoms related to PTE but did not show DVT symptoms such as calf tenderness, swelling, or Homan's sign. On DVT and PTE computed tomography, there were multifocal PTEs in the distal portion of the main and segmental branches of both pulmonary arteries (Fig. 3). There were focal DVTs of the left deep femoral vein, as well as small DVTs in the left popliteal and calf veins (Fig. 4). We immediately referred the patient to the respiratory internal medicine and vascular surgery department for management. After 3 days of low-molecular-weight heparin 1 mg/kg every 12 hours, treatment was changed to an oral drug, Pradaxa (dabigatran, Boehringer Ingelheim GmbH, Ingelheim, Germany) 150 mg bid and used continuously for 6 months.

### 2.4 Follow up and clinical outcomes

There were no DVT findings on DVT sonography at 3 and 6 months postoperative. There were no PTE findings on DVT and PTE CT at 6 months postoperative. The patient has not complained of symptoms related to PTE or DVT at 6 months after the operation, has returned to work, and is living without discomfort.

## 3 Discussion

The most important findings of this study were symptomatic massive PTE and proximal DVT of the femoral vein after arthroscopic meniscus repair surgery. In this case, treatment was successful without any specific complications due to timely intervention.

In general, arthroscopic knee surgery is a low-risk surgery, so prophylactic thromboprophylaxis is not generally required.^[10–12]^ Bushnell et al^[13]^ reported a DVT incidence of 0% to 17.9% after knee arthroscopy surgery. Jaureguito et al^[14]^ confirmed a rate of detectable DVT of 0.24% on ultrasonography. Felcher et al^[15]^ demonstrated a DVT rate of 0.16%, PTE 0.14%, and VTE 0.3% after podiatric surgery. Maletis et al^[8]^ reported an incidence of symptomatic VTE following knee arthroscopy of 0.40%, a relatively low rate; the incidence of symptomatic DVT was 0.25% and that of symptomatic PTE was 0.17%. Hetsroni et al^[7]^ reported a less than 1% rate of symptomatic PTE after arthroscopic surgery. Ye Sun et al^[16]^ investigated VTE after arthroscopic surgery, including not only simple arthroscopic surgery, but also ligament surgery, through venography. The incidence of DVT was 14.9%, proximal DVT 2.4%, and distal DVT 12.5%. In total, 3.7% of cases were symptomatic DVT and the rest were asymptomatic. There were no cases of PTE. Of the 537 patients, 210 underwent simple surgery including meniscus repair, of which the VTE frequency was 9.0%, proximal DVT 2.4%, and distal DVT 6.7%. Although the frequency of VTE is very rare, it is life threatening.

In a study on the risk factors of VTE occurring after arthroscopic surgery, Hetsroni et al reported that risk factors were age 40 years or older, operation time 90 minutes or longer, female sex, and a history of cancer.^[7]^ Ye Sun et al^[16]^ demonstrated that sex, BMI, operation time, and duration of tourniquet application were not risk factors, while age and ligament surgery were risk factors for DVT. Delis et al^[17]^ reported an increase in VTE with the presence of previous DVT and 2 or more risk factors for DVT, including age 65 years of age or older, chronic venous insufficiency, BMI exceeding 30, tourniquet time >30 minutes, and hormone therapy. In our case, the patient had 2 of the risk factors suggested above: BMI exceeding 30 and tourniquet time exceeding 30 minutes.

Caprini et al^[18]^ presented a scoring system that could predict VTE after surgery and assigned scores to low, moderate, high, and highest risk factors. Our patient was obese (BMI >25), which corresponded to a low risk factor in the Caprini scoring system, for a total of 1 point. In addition, moderate risk factors such as arthroscopic surgery, major surgery for more than 45 minutes, and immobilization for less than 1 month were added for a total of 6 points. There were no high or highest risk factors, so the total score was 7 points.^[19]^ According to previous studies,^[19]^ a Caprini score of 7 was associated with 8.84-fold higher risk of VTE than a Caprini score of 0 to 2.

The American College of Chest Physicians provided guidelines for VTE, but thromboprophylaxis was not recommended for arthroscopic knee surgery with no prior VTE history.^[20]^ Other randomized studies also indicated that thromboprophylaxis was not justified in arthroscopic knee surgery. Accordingly, the patient in this study did not undergo thromboprophylaxis before or after surgery but developed massive PTE and DVT after surgery.^[21,22]^

## 4 Conclusion

We report a rare case of massive PTE and DVT in the proximal and distal portions after arthroscopic meniscus surgery in a 32-year-old man. The frequency of VTE is very low after arthroscopic meniscus surgery, but it represents a life-threatening event. Our patient had risk factors for VTE including obesity, surgery time of ∼60 minutes, and immobilization. Although arthroscopic meniscus surgery is relatively safe, evaluation of risk factors for VTE should be performed before and after surgery, and appropriate thromboprophylaxis should be provided when necessary.

## Author contributions

**Conceptualization:** Man Soo Kim.

**Data curation:** Sang Hyun Jeon, Geon Ho Kwon, Man Soo Kim.

**Formal analysis:** Sang Hyun Jeon, Man Soo Kim.

**Funding acquisition:** Man Soo Kim.

**Investigation:** Man Soo Kim.

**Methodology:** Man Soo Kim.

**Project administration:** Man Soo Kim.

**Resources:** Man Soo Kim.

**Software:** Man Soo Kim.

**Supervision:** Man Soo Kim.

**Validation:** Man Soo Kim.

**Visualization:** Man Soo Kim.

**Writing – original draft:** Sang Hyun Jeon.

**Writing – review & editing:** Man Soo Kim.
